# Improvement of CO_2_-DIAL Signal-to-Noise Ratio Using Lifting Wavelet Transform

**DOI:** 10.3390/s18072362

**Published:** 2018-07-20

**Authors:** Chengzhi Xiang, Ge Han, Yuxin Zheng, Xin Ma, Wei Gong

**Affiliations:** 1 School of Remote Sensing & Geomatics Engineering, Nanjing University of Information Science and Technology, Nanjing 210044, China; xcz0726@nuist.edu.cn; 2 School of Remote Sensing and Information Engineering, Wuhan University, Wuhan 430079, China; 3 School of Geodesy and Geomatics, Wuhan University, Wuhan 430079, China; zhyx@whu.edu.cn; 4 Electronic Information School, Wuhan University, Wuhan 430072, China; maxinwhu@gmail.com; 5 State Key Laboratory of Information Engineering in Surveying, Mapping and Remote Sensing, Wuhan University, Wuhan 430079, China; weigong@whu.edu.cn

**Keywords:** differential absorption lidar, CO_2_ monitoring, high-quality signal acquisition, lifting wavelet transform

## Abstract

Atmospheric CO_2_ plays an important role in controlling climate change and its effect on the carbon cycle. However, detailed information on the dynamics of CO_2_ vertical mixing remains lacking, which hinders the accurate understanding of certain key features of the carbon cycle. Differential absorption lidar (DIAL) is a promising technology for CO_2_ detection due to its characteristics of high precision, high time resolution, and high spatial resolution. Ground-based CO_2_-DIAL can provide the continuous observations of the vertical profile of CO_2_ concentration, which can be highly significant to gaining deeper insights into the rectification effect of CO_2_, the ratio of respiration photosynthesis, and the CO_2_ dome in urban areas. A set of ground-based CO_2_-DIAL systems were developed by our team and highly accurate long-term laboratory experiments were conducted. Nonetheless, the performance suffered from low signal-to-noise ratio (SNR) in field explorations because of decreasing aerosol concentrations with increasing altitude and surrounding interference according to the results of our experiments in Wuhan and Huainan. The concentration of atmospheric CO_2_ is derived from the difference of signals between on-line and off-line wavelengths; thus, low SNR will cause the superimposition of the final inversion error. In such a situation, an efficient and accurate denoising algorithm is critical for a ground-based CO_2_-DIAL system, particularly in field experiments. In this study, a method based on lifting wavelet transform (LWT) for CO_2_-DIAL signal denoising was proposed. This method, which is an improvement of the traditional wavelet transform, can select different predictive and update functions according to the characteristics of lidar signals, thereby making it suitable for the signal denoising of CO_2_-DIAL. Experiment analyses were conducted to evaluate the denoising effect of LWT. For comparison, ensemble empirical mode decomposition denoising was also performed on the same lidar signal. In addition, this study calculated the coefficient of variation (CV) at the same altitude among multiple original signals within 10 min and then performed the same calculation on the denoised signal. Finally, high-quality signal of ground-based CO_2_-DIAL was obtained using the LWT denoising method. The differential absorption optical depths of the denoised signals obtained via LWT were calculated, and the profile distribution information of CO_2_ concentration was acquired during field detection by using our developed CO_2_-DIAL systems.

## 1. Introduction

At present, the occurrence of global warming has been further confirmed by the fifth working report of the International Panel of Climate Change [1]. In response to this global challenge, many countries signed the Paris Agreement at the World Climate Conference on 12 December 2015, thereby setting the long-term goal that global temperature will be controlled to rise within 2 °C compared with the industrialized period and realizing balance between carbon emission and sequestration by 2050. The continued increase of CO_2_ concentration, as the major positive radiative forcing factor, has exerted a huge impact on global climate. However, the temporal and spatial distributions of global CO_2_ remain largely uncertain. In particular, the vertical profile information of CO_2_ concentration is still lacking, which restricts an in-depth understanding of the carbon cycle. The precise monitoring of the vertical distribution of CO_2_ concentration is currently an important and challenging scientific issue [2,3,4]. As a representative of active detection technology, differential absorption lidar (DIAL) is widely used in probing atmospheric CO_2_ given that it cannot only measure CO_2_ column but can also provide vertical profiles of CO_2_ concentration distribution. To date, multiple sets of CO_2_-DIAL systems have been established by developed countries, such as the United States, Germany, and Japan [5,6,7,8,9,10].

A set of ground-based CO_2_-DIAL systems has developed by our team since 2013, and highly precise long-term laboratory experiments have been conducted. Results with a random error of less than 1% were achieved in horizontal detection experiments [11]. However, the performance suffered from low signal-to-noise ratio (SNR) in the vertical detection experiments due to decreasing aerosol concentrations with respect to altitude according to the result of our experiments in Wuhan and Huainan. In 2017, the CO_2_-DIAL system was transported to Yangbajing, a small town in Tibet with an altitude of 4300 m, and SNR decreased sharply because of the clean air and low atmospheric pressure in the area. Moreover, two beam detection lasers are transmitted for the CO_2_-DIAL system and the inversion process requires a logarithmic ratio of on-line and off-line wavelength signal intensities. Inversion accuracy depends directly on the denoising quality of on-line and off-line wavelength signals, and low SNR causes the superimposition of the final inversion error. In such cases, an efficient signal denoising algorithm is essential for improving the detection distance and inversion accuracy of the ground-based CO_2_-DIAL system.

Numerous methods have been improved to reduce the noise level [12,13,14,15,16]. Multiple-pulse accumulation and averaging is one of the most widely used denoising methods. However, the denoising effect of this method is poor and useful information can be easily lost in the signal. Other low-pass filters, including Fourier transform (FT), empirical mode decomposition (EMD), and traditional wavelet transform (TWT), are widely used in signal denoising and have achieved good effects. Nevertheless, these methods focus on the denoising of echo signals without considering the subsequent application of signals. Although the denoising of on-line and off-line wavelengths improves the SNR of lidar signals, CO_2_-DIAL always loses important information required for differential absorption detection, thereby reducing the accuracy of CO_2_ concentration inversion. The signals of on-line and off-line wavelengths are received simultaneously by CO_2_-DIAL; thus, the difference information produced by CO_2_ absorption between two signals should be retained while improving SNR. Consequently, the aforementioned methods are ineffective for denoising CO_2_-DIAL signals.

This study introduced a denoising method based on lifting wavelet transform (LWT) for the lidar signals of our ground-based CO_2_-DIAL system. The proposed denoising method is an improvement of TWT and a spatial filter applied to each signal as a function of range. It selects different predictive and update functions according to signal characteristics. Consequently, the LWT denoising method can preserve the characteristic information of signals while improving SNR, which makes it suitable for the signal denoising of CO_2_-DIAL. The proposed method plays a significant role in the accurate field detection of CO_2_.

## 2. Principle and System

**Figure 1:**
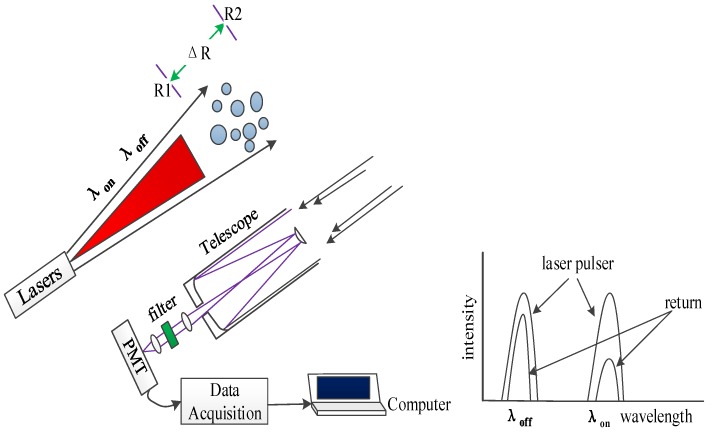
Diagram of ground-based CO_2_-DIAL system.

Figure 1 shows the diagram of the ground-based CO_2_-DIAL [17] system. In the system, two laser pulses with similar wavelengths are transmitted. One laser, called the on-line wavelength (λ_on_), is located at the absorption peak of the detected component to obtain maximum absorption. The other laser, called the offline wavelength (λ_off_), is located near the valley of absorption, which results in minimum absorption.

The lidar equations of the on-line and off-line wavelengths can be written as

(1) $$P(\lambda ,r)=\frac{K\cdot P_{0}\cdot A\cdot c\cdot \frac{\tau }{2}}{r^{2}}\cdot \beta (\lambda ,r)\cdot \exp\left\{-2\cdot \int_{0}^{r}\left[\alpha_{0}(\lambda ,r)+N_{g}(r)\cdot \sigma_{g}(\lambda )\right]dr\right\},$$

where *r* is the detection range, *P*(*λ*,*r*) is the received power of range *r* (*λ* can be on-line and off-line wavelengths), *P*_0_ is the laser output power, *K* is the calibration constant for lidar, *A* is the light area of the receiving telescope, *c* is the speed of light, *τ* is the laser pulse duration, *β*(*λ*,*r*) is the backscatter coefficient of the atmosphere, *α*_0_(*λ*,*r*) is the extinction coefficient of the atmosphere (excluding the trace gas under study), *N_g_*(*r*) is the number density of trace gas, and *σ_g_*(*r*) is the absorption cross section of trace gas [18,19].

In the CO_2_-DIAL system, Equation (1) with the on-line wavelength is divided by that with the off-line wavelength. The number density of the range-resolved CO_2_ can then be derived using the following equation:

(2) $$N_{g}(r)=\frac{1}{2\cdot \Delta r\cdot \left[\sigma_{g}(\lambda_{on})-\sigma_{g}(\lambda_{off})\right]}\ln\left[\frac{P(\lambda_{off},r_{2})\cdot P(\lambda_{on},r_{1})}{P(\lambda_{on},r_{2})\cdot P(\lambda_{off},r_{1})}\right],$$

where *r*_1_ and *r*_2_ are the beginning and end of the integration interval, respectively; and Δ*r* = *r*_2_ − *r*_1_ is the range resolution.

In addition, the concentration of CO_2_ (unit: parts per million, ppm) can be obtained by using the following equation with number density:

(3) $$C=N\cdot \frac{R\cdot T}{N_{A}\cdot P}\times 10^{6},$$

where *N_A_* is the Avogadro constant of approximately $6.022\times 10^{23}{\mathrm{mol}}^{-1}$; *P* and *T* are the pressure and temperature of gas, respectively; and *R* is the ideal gas constant of approximately 8.314 J/(K·mol).

Equation (2) can also be expressed as the following equation of optical depth (OD) and differential absorption OD (DAOD):

(4) $$N_{g}(r)=\frac{DAOD}{2\cdot \Delta r\cdot \Delta \sigma }=\frac{OD(on)-OD(off)}{2\cdot \Delta r\cdot \left[\sigma_{g}(\lambda_{on})-\sigma_{g}(\lambda_{off})\right]}.$$

When the laser wavelength range is small and the time frame is short, several atmospheric parameters change slightly with wavelength, which can be regarded as constant in a DIAL system.

**Figure 2:**
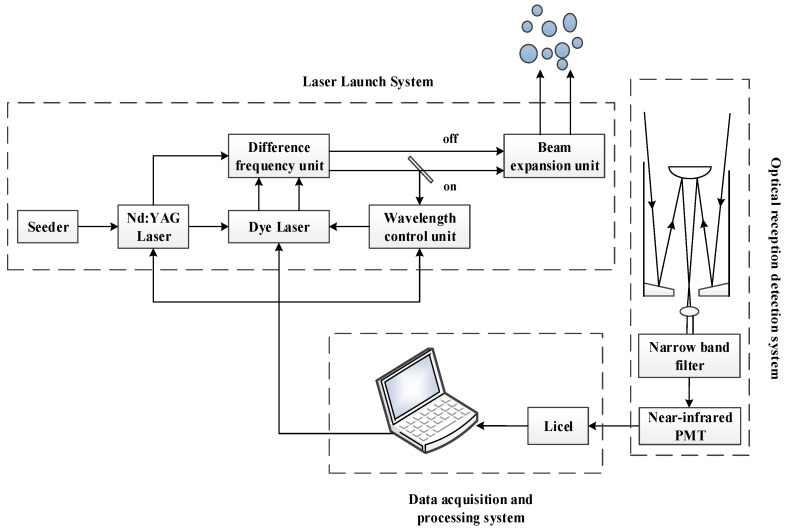
Configuration of our ground-based CO_2_-DIAL system.

**Figure 3:**
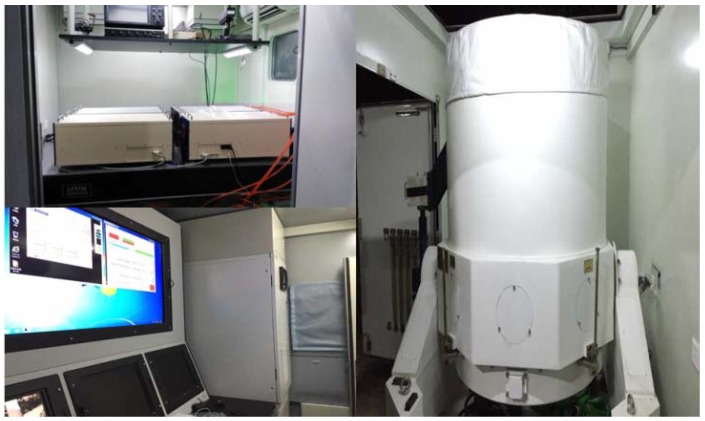
Physical map of our ground-based CO_2_-DIAL system.

**Figure 4:**
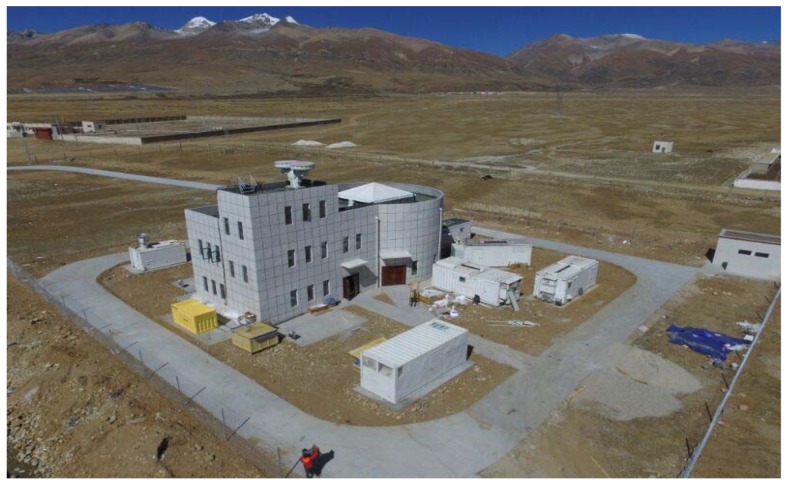
Exterior view of the Yangbajing probing site.

The configuration of our ground-based CO_2_-DIAL system is shown in Figure 2. It is composed of a laser launch system, an optical reception detection system, and a data acquisition and processing system. A pulsed dye laser is used for wavelength modulation in our CO_2_-DIAL system. The target lasers with a wavelength of 1572 nm and a laser repetition frequency of 20 Hz are generated via difference frequency mixing between the fundamentals of the Nd:YAG laser (1064 nm) and the dye laser (~634 nm), which is pumped by the second harmonic of the Nd:YAG laser (532 nm). Consequently, the wavelength of the output laser can be changed by tuning the red laser, the wavelength of which is changed using a stepper motor. In addition, 8% of the pulsed laser light transmitted from the laser system is used as the laser source of the wavelength control unit, whereas the rest is vertically launched into the air. The optical reception detection system is composed of a receiving telescope with an aperture of 1000 mm, a filter bank, and near-infrared photomultiplier tube for echo signal reception, background noise removal, and photoelectric conversion. The data acquisition and processing system is based on a Licel transient recorder for realizing data acquisition and processing functions [11,20,21,22]. The resolution and the sampling frequency of the digitizer is 12-Bit and 20 MHz, respectively. Figure 3 presents the physical map of our ground-based CO_2_-DIAL system, and the exterior view of the Yangbajing site on the Tibetan Plateau is shown in Figure 4.

On the basis of the lidar equations, the quality of a CO_2_ signal will seriously affect the inversion results, particularly for our ground-based CO_2_-DIAL system. In a CO_2_-DIAL system, the concentration of atmospheric CO_2_ is derived from the difference of signals between on-line and off-line wavelengths, and thus, inversion accuracy depends directly on denoising quality. The high-accuracy detection of CO_2_ concentration does not only require an echo signal with high SNR but also the maximum retention of the characteristic information of lidar signals. To improve the detection accuracy of CO_2_-DIAL, a flexible and accurate denoising method based on LWT for the lidar signals of our ground-based CO_2_-DIAL system was proposed.

## 3. Lidar Signal Denoising Based on LWT

The concept of wavelet denoising is derived from stretching and translation, which are conducted on a wavelet basis. First, a signal is transformed into a multiscale signal, and then the wavelet coefficients of the effective signal are extracted at each scale as much as possible while removing the wavelet coefficients that belong to the noise signal. Finally, the signal is reconstructed via inverse wavelet transform. Although TWT is widely used in signal denoising, it requires selecting an appropriate wavelet basis to adapt to different signals. Once a wavelet basis is selected, this basis can no longer be changed during the noise reduction process. This nonadaptive selection of a wavelet basis is a difficult point in TWT application [23].

Although the LWT proposed in this study is based on TWT, it differs from TWT. LWT does not rely on FT, and all its operations are conducted in the time domain. Its wavelet basis is no longer fixed because of the adoption of two independent filters (i.e., predictors and updators) in the lifting wavelet scheme. Each layer decomposition can select the appropriate wavelet basis according to signal characteristics. Furthermore, each step is completely reversible in LWT, thereby providing it with strong adaptability. Consequently, the LWT denoising method is suitable for changeable lidar signals and can effectively preserve the characteristic information of signals [24,25,26].

In the current study, LWT is applied to the denoising of lidar signals based on the signal and noise characteristics of a CO_2_-DIAL system. The basic principle of LWT is to divide a wavelet transform into several steps, with each step conducted according to the characteristics of the signal for an appropriate transformation process. The main steps of lidar signal denoising via LWT are as follows [27,28,29,30,31].

### 3.1. Lifting Wavelet Decomposition (Split–Predict–Update)

(1) Split

This step splits data into two sequences according to odd and even numbers. The two sequences are not intersecting, i.e.,

(5) $$S=(S_{o}(n),S_{e}(n)),S_{o}(n)=x(2k-1),S_{e}(n)=x(2k),k\in z,$$

where *S_o_*(*n*) and *S_e_*(*n*) correspond to the odd and even sequence signals, respectively.

(2) Predict

*P*(*S_e_*(*n*)) is obtained by selecting an appropriate filter function (marked as *P*) and applying it to the even signal. *S_o_*(*n*) can be replaced by *P*(*S_e_*(*n*)) because *S_e_*(*n*) and *S_o_*(*n*) are parity adjacent, and a change between them should be minimal in case of a smooth signal. The advantage of prediction considerably reduces the number of signals for processing. The difference between *S_o_*(*n*) and *P*(*S_e_*(*n*)) [marked as *d*(*n*)] also represents the approximation degree between the parity signals. In this case, *d*(*n*) can be regarded as the high-frequency (HF) component of the signal.

(6) $$d(n)=S_{o}(n)-P(S_{e}(n))$$

(3) Update

The main purpose of this step is to enable the predicted *S_e_*(*n*) to maintain the same global features as the original signal and to obtain the low-frequency (LF) components of the original signal. In the update process, a function *U* is introduced to modify *d*(*n*), and then the updated *d*(*n*) is used to update *S_e_*(*n*) to obtain a new subset of *S*(*K*).

(7) $$S(k)=S_{e}(n)+U(d(n))$$

**Figure 5:**
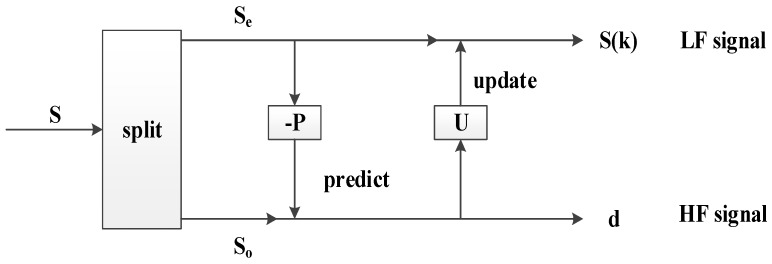
Diagram of lifting wavelet decomposition.

**Figure 6:**
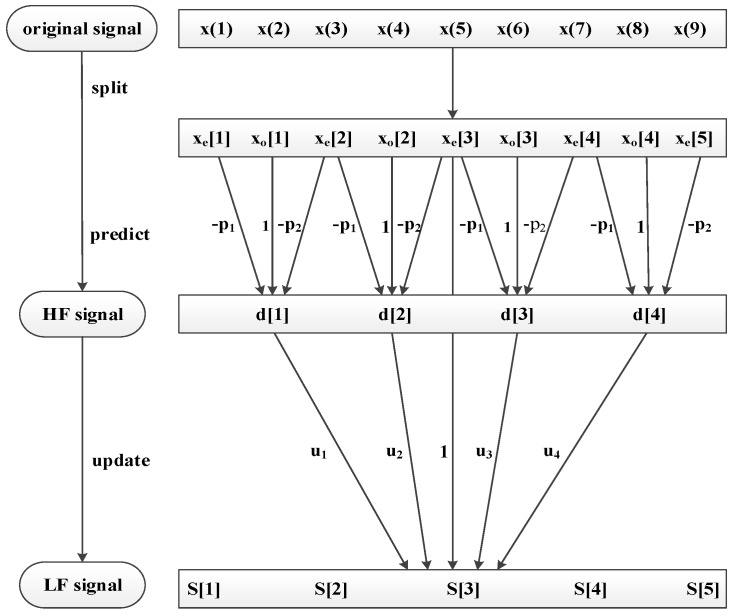
Rendering of lifting wavelet decomposition.

A layer decomposition of *S*(*n*) is achieved through three steps. Furthermore, the same processes of split, predict, and update are performed on the subset. After *j* times lifting wavelet decomposition, *S*(*n*) can be expressed as [*S_n_*_−*j*_, *d_j_*, *d_j_*_−1_, *…*, *d*_1_], where *S_n_*_−*j*_ represents the LF of the signal, i.e., the useful information, whereas [*d_j_*, *…*, *d*_1_] represents the HF of the signal. The principle diagram of signal decomposition is shown in Figure 5. With the assumption that *P =* [*p*_1_, *p*_2_] and *U =* [*u*_1_, *u*_2_, *u*_3_, *u*_4_], the decomposition process of a lifting wavelet is shown in Figure 6.

### 3.2. Threshold Processing

After wavelet decomposition, an appropriate threshold processing method is necessary to deal with the wavelet coefficients obtained through decomposition. At present, hard-field and soft-field value processing are the primary threshold processing methods. In threshold processing, the wavelet coefficients are compared with a given threshold. When the absolute value of the wavelet coefficient is less than that of the threshold, the two processing methods make all the wavelet coefficients zero [32,33,34,35]. However, when the absolute value of the wavelet coefficients is higher than the threshold, the hard-field threshold processing method keeps the coefficients unchanged, whereas the soft-field threshold processing method regards the difference between the wavelet coefficients and the threshold as new wavelet coefficients. In this work, the soft-field threshold processing method is adopted for lidar signal denoising due to its better smoothing effect.

(8) $$d_{\tau }=\mathrm{sgn}(d(n))(\left|d(n)-\tau \right|)=\{\begin{array}{cc} 0, & \left|x\right|\leq \tau \\ d(n)-\tau , & \left|x\right|>\tau \end{array},$$

(9) $$\tau =\sigma \sqrt{2\ln N},\sigma =\frac{1}{0.6745}\cdot Med(\left|d\right|),$$

where *τ* is the threshold, *σ* is the standard deviation estimate of the noise, *N* is the sampling number of the detail signal, and *Med*() is the median function.

### 3.3. Lifting Wavelet Reconstruction (Resume Update–Resume Prediction–Merge)

**Figure 7:**
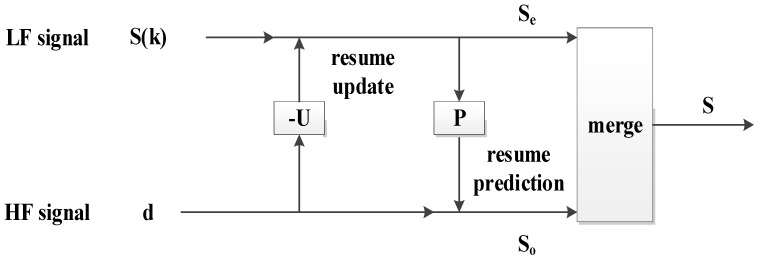
Diagram of lifting wavelet reconstruction.

The last step of denoising is the reconstruction of the wavelet coefficients that were processed by the threshold. In LWT denoising, signal reconstruction is the opposite of signal decomposition. Therefore, a reconstructed signal can be achieved by simply replacing the calculation order and the sign, thereby preventing the failure of signal reconstruction. This feature is another advantage of LWT denoising. The principle diagram of signal reconstruction is shown in Figure 7, and the main processes are described as follows.

(1) Resume update

The even signal *S_e_*(*n*) is resumed from the LF signal *S*(*k*) and the HF signal *d*(*n*).

(10) $$S_{e}(n)=S(k)-U(d(n))$$

(2) Resume prediction

The odd signal *S_o_*(*n*) is resumed by the even signal *S_e_*(*n*) and the HF signal *d*(*n*).

(11) $$S_{o}(n)=P(S_{e}(n))+d(n)$$

(3) Merge

The original signal is resumed by the odd signal *S_o_*(*n*) and the even signal *S_e_*(*n*).

(12) $$S=(S_{o}(n),S_{e}(n))$$

**Figure 8:**
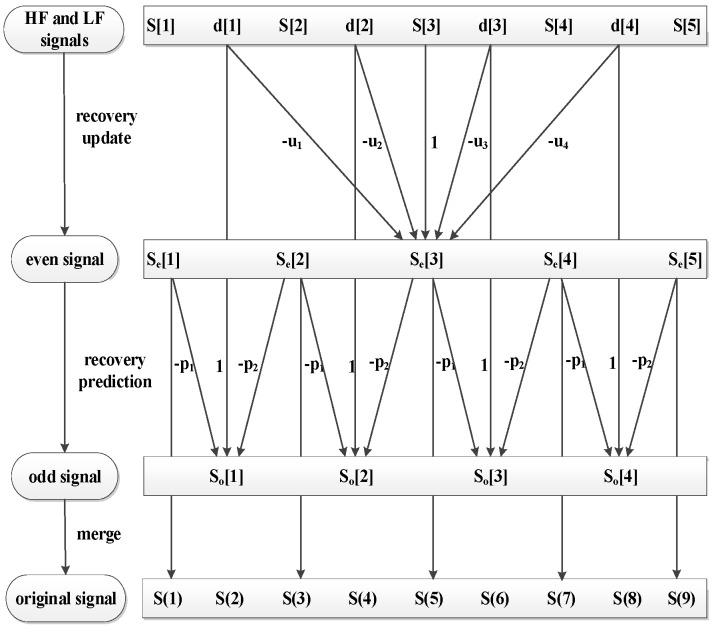
Rendering of lifting wavelet reconstruction.

The lifting wavelet reconstruction process is illustrated in Figure 8.

**Figure 9:**
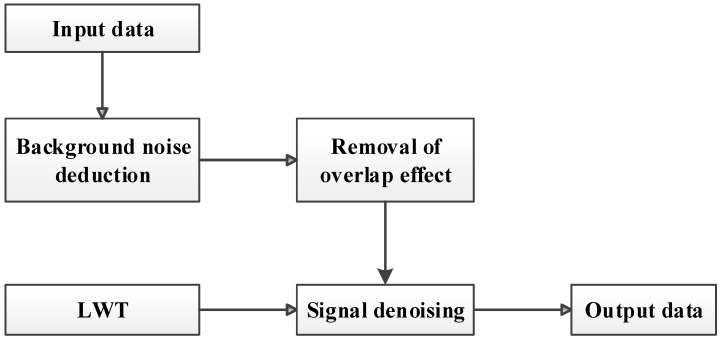
Flowchart of lidar signal denoising via LWT.

Finally, the flowchart of lidar signal denoising via LWT for atmospheric CO_2_ detection is shown in Figure 9.

## 4. Experiment Analysis

LWT is applied to the processing of CO_2_-DIAL signals to evaluate the effect of denoising. The lidar signals, which are collected from 8:00 p.m. to 11:30 p.m. on 30 July 2016 in Huainan, China, are used as the experiment data. The time resolution of our CO_2_-DIAL system is 30 s, the laser transmission frequency is 20 Hz, the resolution and sampling frequency of Licel is 12-Bit and 20 MHz, respectively. The wavelengths of on-line and off-line lasers in our system are 1572.018 nm and 1572.150 nm, respectively. For simplicity, this paper presents only off-line wavelength signals because the echo signals of on-line and off-line wavelengths are similar.

### 4.1. Background Noise Deduction

**Figure 10:**
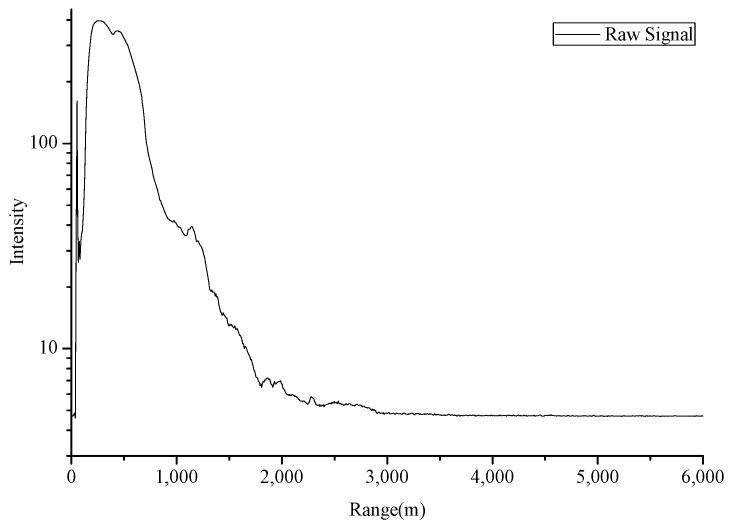
Lidar signal after the subtraction of background noise (30 July 2016).

**Figure 11:**
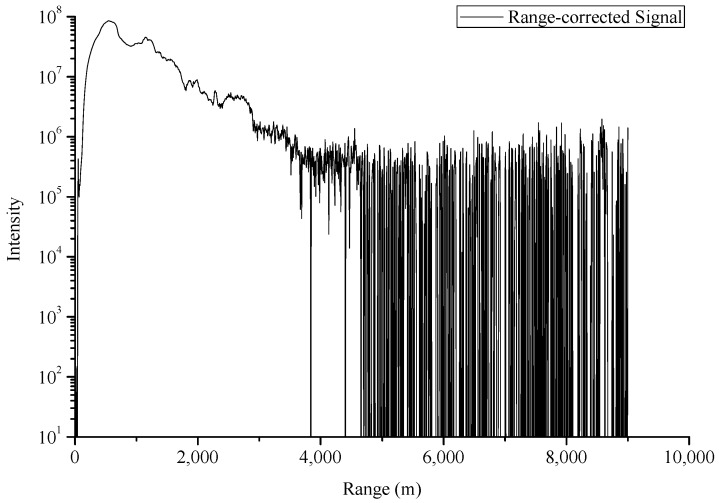
Lidar signal corrected by distance (30 July 2016).

The background noises of lidar signals are mainly composed of two parts: system background noise and sky background light noise. These noises can be measured and removed roughly in a lidar detection system. In our experiments, the process of system background noise removal are as follows: The CO_2_-DIAL system was first operating normally for 5 min with the telescope covered to ensure that there are no scattering signals received by the infrared detector. Then, lidar signals were collected and used to estimate a system noise for subtraction from the subsequent live signal. As to the sky background noise, the signals received by the telescope at high detection area can be considered as the sky background light noise because the laser detector cannot reach this height. Therefore, in our experiment, we calculated the mean value of signal intensity of about 20–22 km altitude range as sky background noise and subtracted them from lidar signals. Figure 10 illustrates the lidar signal after the subtraction of background noise, whereas Figure 11 shows the signal corrected by distance.

It is undeniable that taking these measures cannot remove all the noise, and the random noise still exists. In order to obtain high accuracy vertical profile information of CO_2_ concentration, an effective signal denoising method is still needed to improve the quality of the signal in the subsequent data processing.

### 4.2. Selection of Wavelet Function and Decomposition Scale

**Figure 12:**
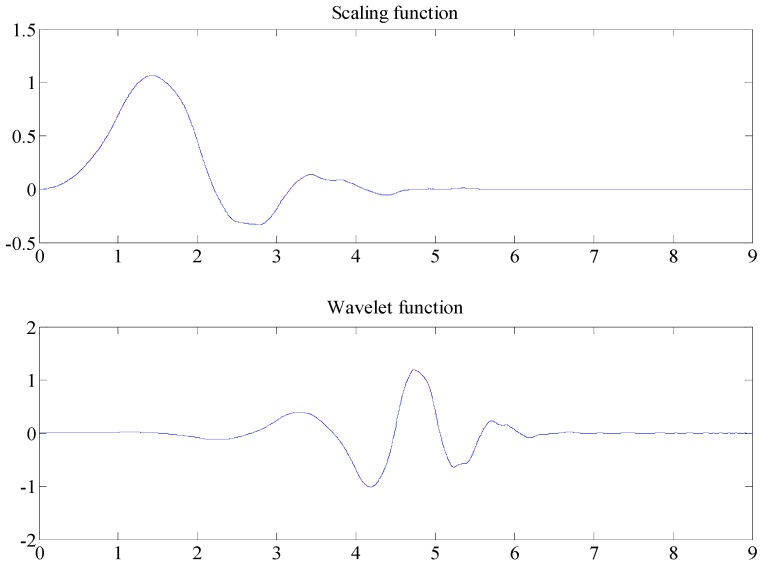
Graph of the db5 wavelet scaling function and wavelet function.

Before denoising, the wavelet function and the decomposition scale are first selected. This selection directly determines the quality of denoising. A comparison of various wavelet functions with lidar signals shows that the db5 wavelet exhibits a high degree of similarity with lidar signals. Figure 12 shows the graph of the db5 wavelet scaling function and the wavelet function. Consequently, this study adopts the db5 wavelet lifting algorithm for lidar signal denoising.

A simulation experiment was conducted to select a suitable wavelet decomposition scale for signal denoising via LWT, which can enable the denoised signal obtained through LWT to preserve the characteristic information of signals while improving SNR. In the experiment, the concentration of CO_2_ was assumed as a fixed value, and then the theoretical lidar signals were obtained by the inversion equation, i.e., Equation (2). The db5 wavelet was used to denoise analog signals at different decomposition levels, and the decomposition scales are one to four layers in turn. After signal denoising is completed, the linear relationship between the original and denoised signals was calculated to evaluate the denoising effect.

**Figure 13:**
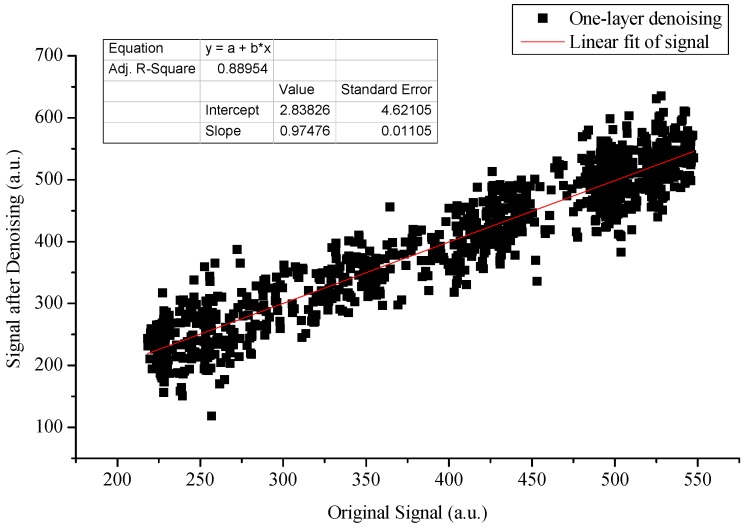

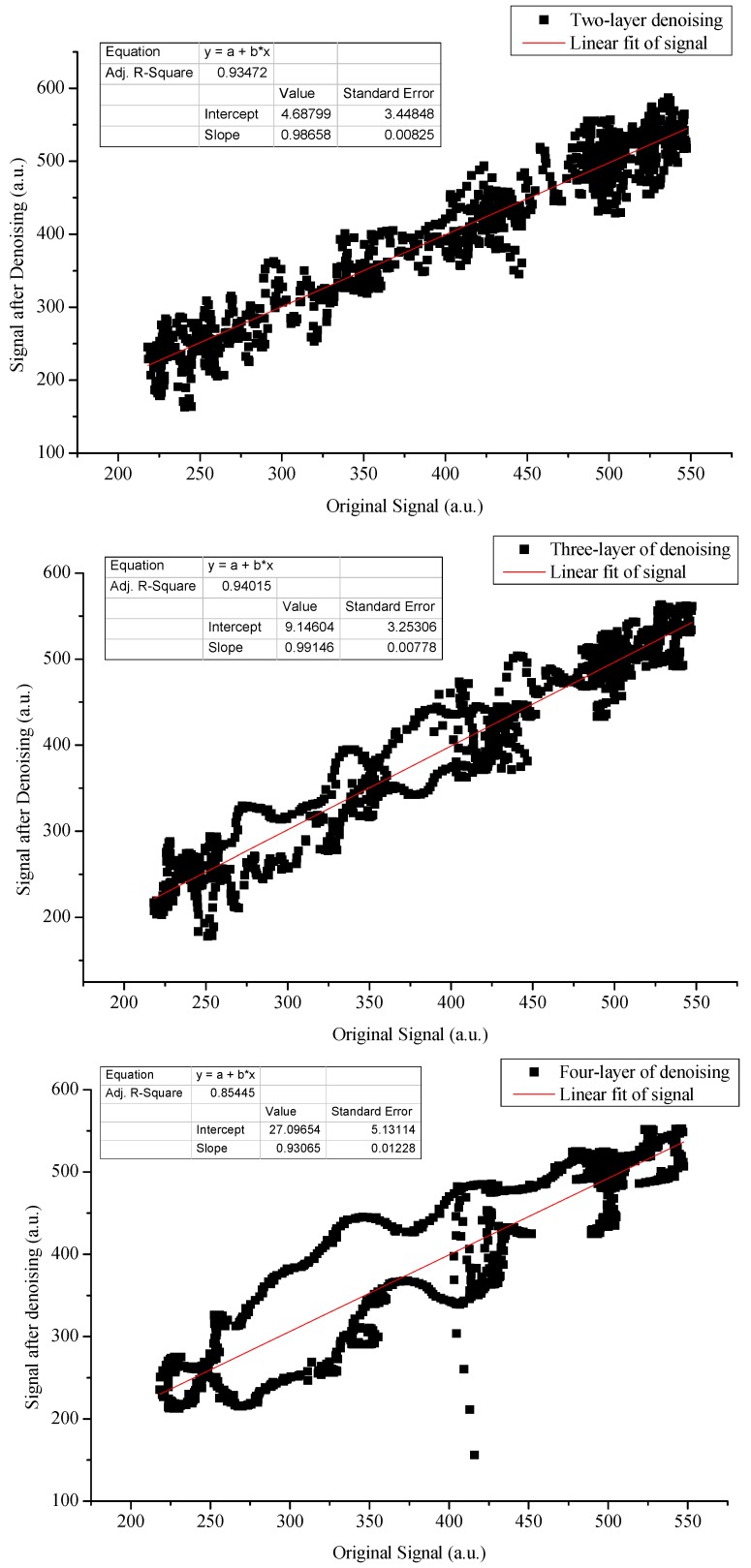
Denoising effect of db5 LWT under different decomposition scales (the decomposition scales are one to four layers in turn).

The results are provided in Figure 13, where the abscissa represents the original signal and the ordinate represents the denoised signal. A good linear relationship exists between the two signals, thereby indicating that the lifting wavelet algorithm exerts a good denoising effect on lidar signals and can maintain the characteristic of the original signal. In addition, the best denoising effect occurs when the decomposition level has three layers according to the linear fitting parameters after the denoising of different decomposition levels. Such effect can effectively preserve the characteristic information of signals.

Table 1 lists the linear fitting parameters between the original signal and the denoised signal under different wavelet decomposition scales. The table shows that when the decomposition scale has three layers, the degree of linear fitting is the highest, which indicates the best denoising effect. After the decomposition of layer three, the denoising effect deteriorates mainly because the signal itself is smoothed away with considerably more useful information as the decomposition scale increases. Therefore, the db5 wavelet function decomposition scale used in the lifting wavelet denoising process in this work has three levels.

### 4.3. Lifting Wavelet Signal Denoising

**Figure 14:**
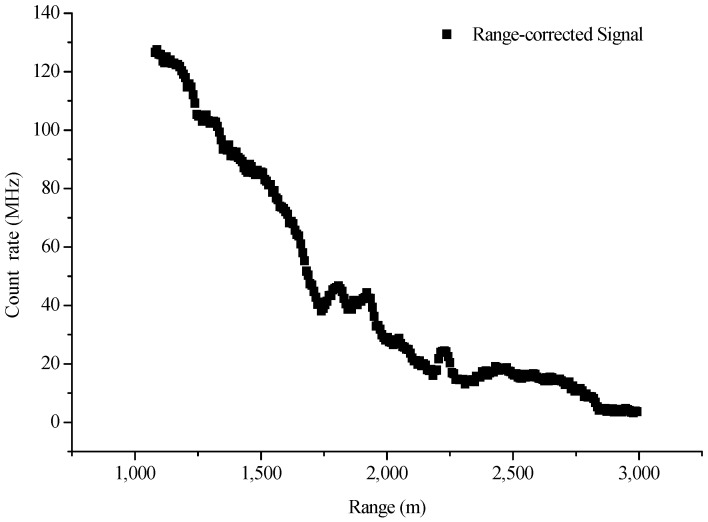
Range-corrected signal at an altitude range of 1000–3000 m (30 July 2016).

**Figure 15:**
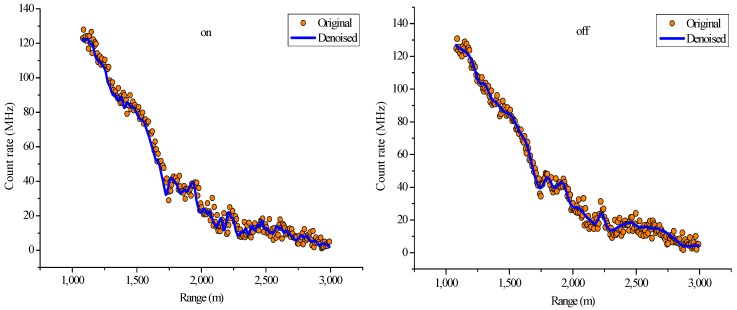
Original and denoised signals via LWT ((**left**) on-line wavelength; (**right**) off-line wavelength).

In this study, lidar signals with an altitude range of 1000–3000 m are selected to conduct wavelet denoising. Figure 14 shows the lidar signals in this region, which is above the atmospheric boundary layer and has a considerably weakened signal intensity, thereby causing low SNR. Therefore, lidar signal denoising is important in this region. On the basis of a pre-period analysis and the selection of a lifting wavelet function and a decomposition level, the mother wavelet of the denoising algorithm in this study is a db5 wavelet, and the decomposition scale has three layers. The denoising effects of the online and off-line wavelength signals are shown in Figure 15.

The figure shows that compared with the original signal, the signal denoised using the lifting wavelet algorithm is considerably smoother while still retaining the basic characteristics of the original signal.

### 4.4. Evaluation of Denoising Effect

For the measured signal, the relative error among echo signals is generally used to evaluate signal quality. The time interval between each signal is extremely short and CO_2_ concentration does not change considerably within such a short time. Thus, the relative error among the signal intensities of echo signals in the adjacent time slot should be minimal at the same detection altitude. However, the relative error increases due to the influence of noise. Therefore, signal quality can be evaluated by calculating the relative error of multiple echo signals at the same altitude over a certain period, i.e., the coefficient of variation (CV).

CV is calculated as follows:

(13) CV=σ/μ,

where *σ* is the standard deviation of the echo signal intensity at the same altitude, and *u* is the mean value of data.

To evaluate the denoising effect of lifting wavelet, this study calculated the CV at the same altitude among multiple original signals within 10 min and then performed the same calculation on the denoised signal.

**Figure 16:**
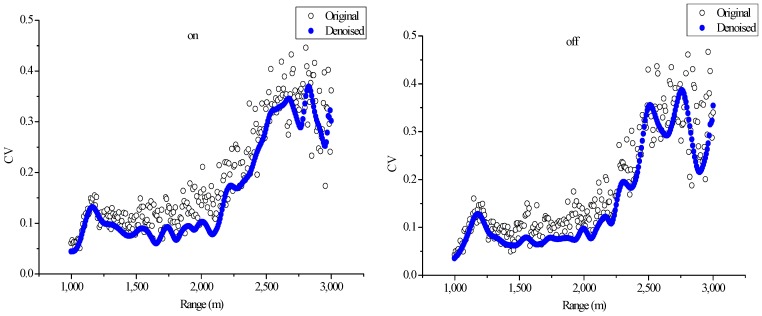
CVs of original signals and denoised signals via LWT ((**left**) on-line wavelength; (**right**) off-line wavelength).

The CVs at different altitudes are shown in Figure 16. The CVs of the on-line and off-line wavelengths are considerably reduced after denoising using the lifting wavelet algorithm, which indicates that the algorithm exhibits good capability for lidar signal denoising. The figure also illustrates that SNR decreases while CV increases at a high altitude.

**Figure 17:**
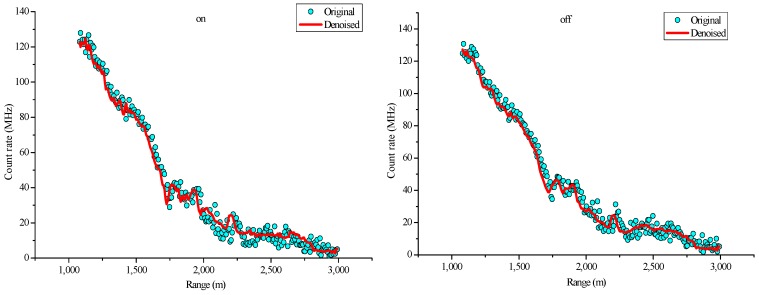
Original and denoised signals using EEMD ((**left**) online wavelength; (**right**) off-line wavelength).

To prove the signal denoising effect of lifting wavelet, the ensemble empirical mode decomposition (EEMD) denoising is conducted on the same lidar signal in this study. EEMD, which is an improvement of EMD, was proposed by Huang et al. to solve the modal aliasing problem of EMD. It decomposes a signal into a series of intrinsic mode functions, which are arranged from high to low frequencies at different time scales. Noise generally exists in high-frequency components; thus, signal noise reduction can be achieved by removing high-frequency components [13,36,37]. EEMD does not require pre-selection of the basis function. This method decomposes based on the signal itself, which makes it adaptable; it also exhibits good denoising effect on lidar signals [38,39]. The denoising effect of the lifting wavelet method proposed in this work can be measured well by comparing with the result of EEMD denoising. The results are presented in Figure 17.

**Figure 18:**
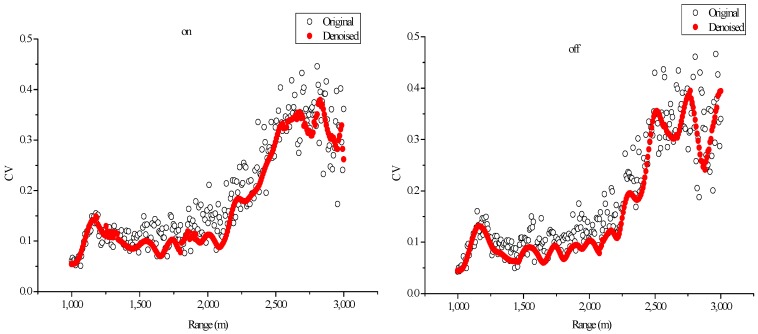
CVs of the original signals and denoised signals using EEMD ((**left**) on-line wavelength; (**right**) off-line wavelength).

Figure 18 shows the CVs of the intensity of multiple signals at the same height. The average values of CV calculated from the original signal and the denoised signals using the two methods are listed in Table 2. The lifting wavelet algorithm proposed in this study is demonstrated to be capable of achieving better denoising effect than EEMD.

**Figure 19:**
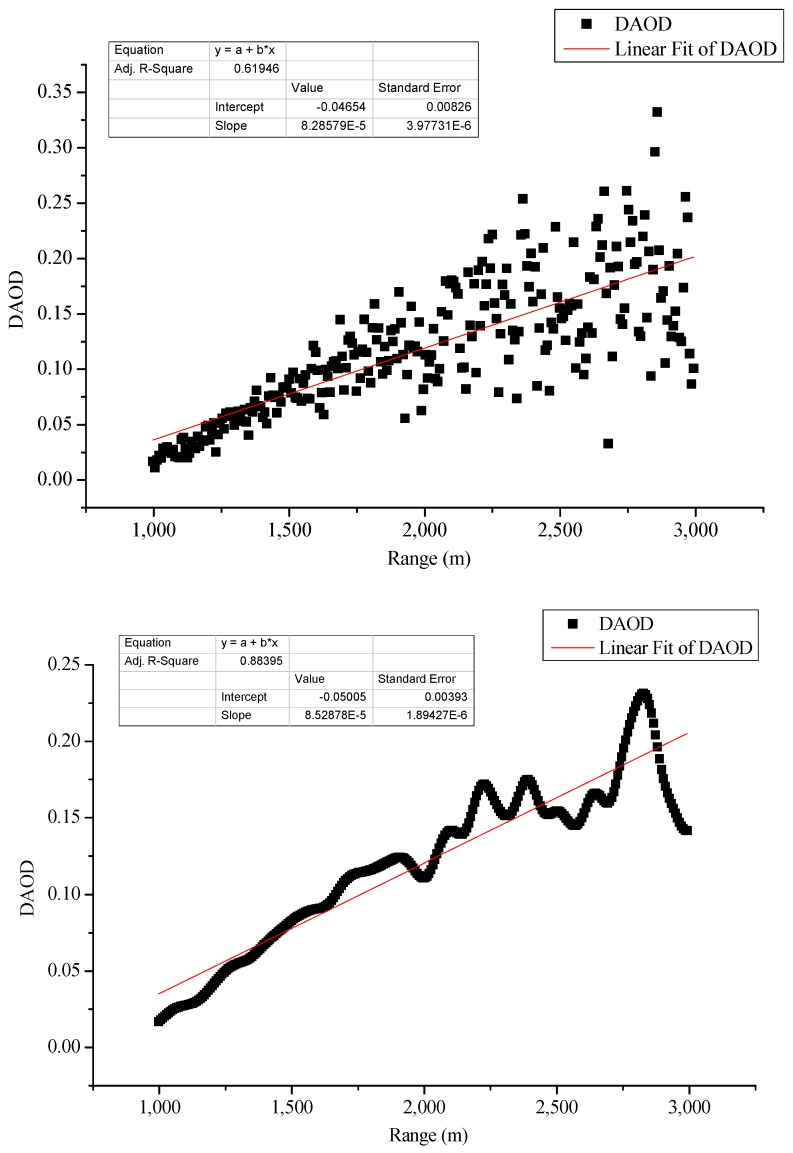
Calculated DAOD of the signal after denoising via LWT. The above is the DAOD of original signal, and the below is the DAOD of denoised signal via LWT.

In addition, the DAOD of lidar signal after denoising via LWT is calculated. The results are presented in Figure 19, and the linear fitting parameters are listed in Table 3.

The DAOD singular value of the signal caused by noise is eliminated after denoising via LWT, and the linear fitting effect is also considerably improved. These results show that the LWT algorithm proposed in this study can effectively improve the quality of echo signals. The LWT denoising method cannot only improve SNR but can also preserve the characteristic information of signals, which is favorable for the subsequent detection of CO_2_ using our CO_2_-DIAL system.

**Figure 20:**
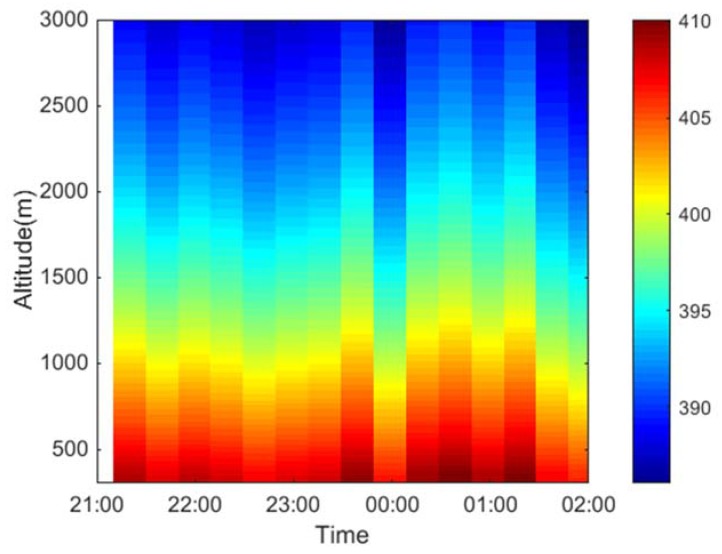
Pseudo-color map of CO_2_ vertical concentration detected using our CO_2_-DIAL system.

Finally, long-term field experiments were conducted in Huainan, China, and the profile distribution information of CO_2_ concentration was obtained using our CO_2_-DIAL system, as shown in Figure 20.

## 5. Conclusions

In this study, a denoising method based on LWT was proposed to acquire high-quality echo signals of our ground-based CO_2_-DIAL system in field exploration. The LWT denoising method can preserve the characteristic information of signals while improving SNR, which is highly significant for the accurate field detection of CO_2_. Experiment analyses were conducted to evaluate the denoising effect of LWT. For comparison, EEMD denoising was also conducted on the same lidar signal. Moreover, this study calculated the CVs at the same altitude among multiple original signals within 10 min, and then performed the same calculation on the denoised signal. The respective average values of CV calculated from the denoised signals of LWT and EEMD were 0.1637 and 0.1751 for on-line wavelength and 0.1508 and 0.1645 for off-line wavelength, respectively. The results show that the CVs of multiple signals at the same height were reduced after denoising via LWT, and the average CV value calculated from the denoised signals of LWT was smaller than that calculated from the denoised signals of EEMD, thereby indicating that LWT achieved better denoising effect than EEMD. In addition, the DAOD values of the original and denoised signals via LWT were calculated. The DAOD singular value of the signal caused by noise was eliminated after denoising by LWT, and the linear fitting effect was considerably improved, with *R*^2^ increased from 0.619 to 0.884, thereby implying that the detection accuracy of CO_2_ was significantly improved. Finally, long-term field experiments were conducted in Huainan, China, and the profile distribution information of CO_2_ concentration was obtained using our CO_2_-DIAL system with the LWT denoising method.

## Figures and Tables

**Table 1:** Denoising evaluation parameters of db5 LWT under different decomposition scales.

	n = 1	n = 2	n = 3	n = 4
***k***	0.975	0.987	0.991	0.931
***R*^2^**	0.890	0.935	0.940	0.854

**Table 2:** Average values of CV calculated by the original and denoised signals.

	Original Signal	EEMD Denoised Signal	LWT Denoised Signal
**On-line**	0.2115	0.1751	0.1637
**Off-line**	0.1940	0.1645	0.1508

**Table 3:** Linear fitting parameters of the original and denoised signals.

	Original Signal	LWT Denoised Signal
***k***	8.286E-5	8.529 E-5
***R*^2^**	0.619	0.884
